# Acrosomal Status and PARP‐1 Nuclear Markers Could Improve Discrimination of Potential Fertility in Good‐Quality Boar Semen Doses

**DOI:** 10.1111/rda.70145

**Published:** 2025-11-12

**Authors:** Raquel Ausejo‐Marcos, Sara Miguel‐Jiménez, M. Teresa Tejedor, Belén Gómez‐Giménez, Cristina Soriano‐Úbeda, Felipe Martinez‐Pastor, Noelia Mendoza, Alejandro Vicente‐Carrillo, William Fernando Hurtado, Celia Ávila Holguín, Bernardino Moreno, María Victoria Falceto

**Affiliations:** ^1^ Department of Biotechnology R&D Magapor S.L Ejea de los Caballeros Spain; ^2^ Department of Research and Development CiencIAnova Magapor Zaragoza Spain; ^3^ Department of Anatomy, Embryology and Genetics, CIBERCV, Faculty of Veterinary Medicine University of Zaragoza Zaragoza Spain; ^4^ Institute of Animal Health and Cattle Development (INDEGSAL) University of León León Spain; ^5^ Department of Veterinary Medicine, Surgery, and Anatomy (Animal Medicine and Surgery) University of León León Spain; ^6^ Department of Molecular Biology (Cell Biology) University of León León Spain; ^7^ Department of Animal Pathology, Obstetrics and Reproduction, Faculty of Veterinary Medicine University of Zaragoza Zaragoza Spain

**Keywords:** boar semen, semen analysis, seminal plasma, swine reproduction

## Abstract

Reproductive efficiency in boars partly depends on semen quality. However, it is challenging to predict sperm fertility once acceptable quality endpoints have been met. This study aims to establish a link between different semen quality parameters and farm fertility outcomes in semen doses selected for commercial use. We analysed 105 ejaculates from 15 adult Pietrain boars, extended into 45 mL artificial insemination (AI) doses. A total of 605 sows were inseminated (40.1 ± 7.8 females/boar) and fertility, farrowing rate and prolificacy data were recorded. Sperm evaluation included sperm plasma analysis, kinematics, morphology, viability, acrosome integrity, apoptotic‐like changes, mitochondrial activity and DNA damage (DNA fragmentation poly‐ADP ribose polymerase 1, PARP1, together with its product PAR and its cleaved form cPARP). The sperm membrane and acrosome were evaluated using the short hypo‐osmotic swelling test (sHOST) and the osmotic resistance test (ORT). Fertility and farrowing rates exceeded 94%, with an average of 20.18 ± 2.03 piglets born/litter (average born alive: 87.75% ± 5.61%). Negative correlations were found between damaged acrosomes and ultrasound fertility (ρ = −0.240, *p* = 0.021), farrowing rate (ρ = −0.244; *p* = 0.019), and total born (*r* = −0.304; *p* = 0.003). Average born alive was positively correlated with plasma seminal concentrations of protein (*r* = 0.273; *p* = 0.009), fructose (*r* = 0.243; *p* = 0.024) and cathepsine B (ρ = 0.257; *p* = 0.029) but negatively correlated with apoptosis and DNA damage nuclear markers cPARP (*r* = −0.295, *p* = 0.005) and PAR (*r* = −0.209, *p* = 0.049). However, regression models only showed significant results for predicting the total born from damaged acrosomes and those born alive from cPARP, although the coefficients of determination were very low. Since semen quality and fertility were good, most parameters did not affect fertility outcomes. In agreement with previous studies, acrosomal damage would be a reliable predictor of reproductive outcomes, whereas cPARP would show potential as a novel biomarker.

## 1 Introduction

Conventional sperm quality assessment detects sub‐fertile boars and ensures sperm quality of semen doses (Ruiz‐Sánchez et al. 2006). However, their predictive value is limited, so other cell and molecular parameters have been tested using flow cytometry (Boe‐Hansen and Satake 2019). The chromatin status shows promise for discriminating among boars and ejaculates (Ausejo et al. 2022; Mateo‐Otero et al. 2022) and novel parameters such as poly (ADP‐ribose) polymerase 1 (PARP1) activity (poly (ADP‐ribose)), PAR, (presence) or its cleaved form (cPARP) have been proposed for this purpose (Casao et al. 2015).

Additionally, seminal plasma (SP) influences sperm function and reproductive success. It contains a complex mixture of proteins, antioxidants, hormones and other molecules that influence sperm function, survival and fertilisation (Dyck et al. 2011; Rodriguez‐Martinez et al. 2021), as well as modulating the female reproductive tract (Parrilla et al. 2020). Thus, combining the analysis of sperm parameters and seminal plasma could improve the predictive power of semen testing (Llavanera 2024).

Using data from commercial farms, this study explores the relationship between sperm variables and SP components with fertility and prolificacy.

## 2 Material and Methods

This study followed ARRIVE guidelines (Kilkenny et al. 2010), Council Directive 2008/120/EC (minimum standards for the protection of pigs) and Directive 2010/63/EU (protection of animals used for scientific purposes). The Ethics Committee of the University of Zaragoza approved the study (PI36/24).

### 2.1 Animals

Fifteen young healthy Pietrain boars aged 681.4 ± 67.52 days were included (genetic lines Nucléus: 10, weekly sperm collection, January–March 2022; and Batallé, four‐day collection, March–April 2022). Boar crates were at least 6 m^2^ with *ad libitum* water. Animals were fed once a day (commercial diet) and lighting and temperature were controlled (20°C–22°C).

### 2.2 Semen Collection and Seminal Doses

The double‐gloved‐hand technique was used for semen collection, discarding pre‐sperm and gel fractions. Semen was tested by CASA (Computer‐Assisted Sperm Analysis) in the same center to exclude ejaculates with < 70% total motility and > 25% abnormal morphology. Magapor SL established additional exclusion criteria as described previously (Ausejo‐Marcos et al. 2024). Therefore, 105 ejaculates were accepted for the preparation of seminal doses (3–9 ejaculates/boar for Nucléus and 6 ejaculates/boar for Batallé genetic lines).

The ejaculates were diluted to 45‐mL seminal doses with a high‐performance commercial extender (Duragen; Magapor SL, Ejea de los Caballeros, Spain) at 2.9 × 10^7^ cells/mL and kept at 15°C–17°C. For evaluation of conservation, two doses were kept in the AI stud for 5 days. Evaluations were performed in an external laboratory (CiencIAnova, Magapor AIE, Zaragoza, Spain), which received two other doses and undiluted ejaculate for assessing sperm parameters and seminal plasma isolation. The remaining doses were sent to the sow farms for inseminations.

### 2.3 Sperm Plasma Analyses

Ejaculates were centrifuged (1500 × g, 10 min) to obtain SP which was aliquoted and kept frozen (−80°C) until analysis.

SP analyses were carried out using commercial kits according to the manufacturer's instructions. Protein concentration was assessed with the Micro BCA Protein Assay Kit (ThermoFisher, Waltham, MA) and fructose, zinc and citrate concentrations by D‐Glucose/D‐Fructose, BROMO‐PAPS and Citrate/Citric Acid kits with an A15 Access Analyser (Biosystems, Barcelona, Spain). Enzymatic activities were assessed colorimetrically: Superoxide Dismutase (SOD) Colorimetric Kit and mammalian β‐Galactosidase Assay Kit (Thermo Fisher Scientific); Pig Epididymal secretory GPX ELISA Kit (MyBioSource Inc., San Diego, CA) for glutathione peroxidase 5 (GPX5); Catheptsin B Activity Assay Kit for Cathepsin B and Alkaline Phosphatase Assay Kit for alkaline phosphatase (ALP) (Abcam, Cambridge, UK).

### 2.4 Sperm Assessment

#### 2.4.1 Motility and Concentration

Motility analysis was performed with a commercial boar‐specific CASA (Magavision ELITE, Magapor). Samples were pre‐warmed (15 min, 37°C) and 3.5 μL were placed in a 20‐μm chamber (Magapor SL), recording at least 500 cells (60 fps, 1 s) at ×10pHN. The CASA provided total (TMot) and progressive motility (PMot), fast, intermediate, slow and static spermatozoa, curvilinear (VCL, μm/s), straight line (VSL, μm/s) and average velocity path (VAP, μm/s), linearity (LIN, VSL/VCL,%), straightness (STR, VSL/VAP, %), wobble (WOB, VAP/VCL, %), amplitude of lateral head displacement (ALH, μm) and beat cross frequency (BCF, Hz).

#### 2.4.2 Sperm Morphology and Acrosome Status

Sperm cells were stained (1:1) with an eosin‐nigrosine solution (Magapor SL) and examined under a bright‐field microscope at ×1000 for sperm morphology. At least 200 spermatozoa were counted per slide and the percentage of sperm with intact acrosomes and total abnormal forms (AF) were recorded. Spermatozoa were considered abnormal when they had head, midpiece or tail defects or cytoplasmic droplets.

#### 2.4.3 Short Hypo‐Osmotic Swelling Test and Short Osmotic Resistance Test

Sperm membrane functionality and acrosomal resistance were assessed using the short hypo‐osmotic swelling test (sHOST) and the short osmotic resistance test (sORT) (Rota et al. 2000) as described previously (Pérez‐Llano et al. 2001). At least 200 spermatozoa were counted per slide. Sperm with curled tails after sHOST and intact acrosomes (acro%) after sORT were expressed as proportions.

#### 2.4.4 Flow Cytometry Analysis

The membrane and acrosomal integrity, apoptosis and mitochondrial activity were assessed with a BD Accuri C6 flow cytometer with BD software (Becton Dickinson, Madrid, Spain), equipped with 2 laser sources (blue, 488 nm and red, 640 nm) and 4 fluorescence detectors (blue line: FL1 533/30, FL2 585/40; FL3 670LP; red line: FL4 675/25). At least 20,000 events were recorded. Debris was excluded based on forward scatter (FS) and side scatter (SS) characteristics.

#### 2.4.5 Sperm Membrane and Acrosome Integrity

Samples (5 × 10^6^ cells/mL) were incubated (15 min, 37°C in darkness) with 16 μM propidium iodide (PI) and 15 μg/mL FITC‐PNA (peanut agglutinin) (Merck, Darmstadt, Germany) and analysed by flow cytometry (FL1 PNA and FL2 PI), recording the percentages of viable (PI‐) and acrosome‐damaged spermatozoa (PNA+).

#### 2.4.6 Apoptosis and Mitochondrial Activity

Samples (5 × 10^6^ cells/mL) were incubated (15 min, 37°C in darkness) with 40 nM YO‐PRO‐1 and 125 nM MitoTracker deep red (ThermoFisher) and analysed by flow cytometry (FL1 YO‐PRO‐1 and FL4 MitoTracker), recording the percentages of early apoptotic (YO‐PRO‐1+) and high‐mitochondrial activity spermatozoa (MitoTracker+) (Mendoza et al. 2013; Nerin et al. 2018).

#### 2.4.7 Sperm Chromatin Assessment

The Sperm Chromatin Structure Assay (SCSA) was used to assess DNA fragmentation (Evenson 2022). Samples were diluted with TNE buffer (0.01 M Tris–HCl, 0.15 M NaCl, Na_2_EDTA 1 mM; pH 7.4) up to 2 × 10^7^ mL^−1^ and stored at −80°C until processing in the reference laboratory (INDEGSAL, León, Spain). After thawing, 100 μL were treated with 400 μL of acid‐detergent solution (0.1% Triton X‐100, 150 mM NaCl and 80 mM HCl; pH 1.2) for 30 s followed by 1.2 mL of staining buffer (6 μg/mL acridine orange, 100 mM citric acid, 200 mM Na_2_HPO_4_, 1 mM Na_2_EDTA, 150 mM NaCl; pH 6.0). Samples were run through a FACScalibur flow cytometer (Becton Dickinson) with CellQuest v5 software. The blue laser excited acridine orange, collecting the fluorescence emission with filters 525/50 (green) and 665/730 (red). At least 2000 spermatozoa were counted within 3 min. DNA fragmentation (%DFI) was calculated as the proportion of spermatozoa with high DFI (red/total fluorescence). Sperm nuclear compaction was estimated by high DNA stainability (%HDS) as spermatozoa with green fluorescence above 650 on a 1024 scale.

PARP‐1 and its derivatives, cPARP and PAR, were detected using immunostaining with validated antibodies (Abcam) as described previously, assessing samples with a Cytek Aurora spectral cytometer (Cytek Biosciences, Amsterdam, The Netherlands) using the SpectroFlo v.3.3.0 software. Samples (100 μL) were washed (PBS, 1000 × g 11 min, 4°C) and fixed (4% formaldehyde, 20 min) washed again and incubated with 10 mM dithiothreitol (DTT) (15 min, 37°C) to induce nuclear decondensation. They were then washed and made permeable using a 0.1% Triton solution (0.1% sodium citrate, 30 min on ice) and blocked with 4% BSA in PBS (1 h). Samples were washed and incubated overnight at 4°C with the primary antibodies anti‐PARP1 (436400, ThermoFisher), anti‐cPARP (44‐698G, ThermoFisher) and anti‐PAR (ab14459, Abcam), 1/200 in PBS. Samples were washed and labelled with fluorescent secondary antibodies conjugated with Alexa Fluor: 647 for PARP1 (ab150111, Abcam), 488 for cPARP (ab150081, Abcam), and 568 for PAR (A11041, Invitrogen). After washing, samples were counterstained with 2.7 μM Hoechst 33342 (H342) and assessed (at least 5000 spermatozoa per sample). Debris was gated out as H342‐ and mean fluorescence intensity (MFI) and ratios cPARP/PARP1 and PAR/PARP1 were recorded using Weasel v.3.7 software (Frank Battye, Melbourne, Australia).

### 2.5 Farm Trial: Insemination and Monitoring of Inseminated Sows

Sperm doses were sent to two commercial sow farms (size 2500–2700 sows) in the northeast of Spain and applied by post‐cervical artificial insemination (PCAI, Magapor SL). Heat detection was done using a teaser boar and only sows in heat were inseminated.

A total of 605 DanBred hyperprolific sows were twice inseminated weekly in batches of 40–55 (40.1 ± 7.8 sows/boar). Each female received semen only from the same boar throughout the oestrus. For each ejaculate, fertility and prolificacy data [average ultrasound fertility (%, AUF), farrowing rate (%, AFR), total born (%, ATB) and born alive (%, ABA) were recorded].

### 2.6 Statistical Analysis

Statistical analyses were carried out using IBM SPSS Statistics 26.0 software (IBM Corp., Armonk, NY, USA). Normal distribution was tested by Shapiro–Wilk. Non‐normally distributed variables were transformed (log_10_, square root, or arcsine). Descriptive statistics use mean ± SD (standard deviation). Pearson's (*r*) and Spearman's (ρ; non‐normal variables) correlation coefficients were used to estimate the association between reproductive success and sperm parameters. Multiple regression models were adjusted for the prediction of reproduction success (response variables: AUF, FR, ATB or ABA) from sperm parameters (explanatory variables) with a forward procedure (*p* values for *F* test 0.05 for entering and 0.10 for leaving the model). Only variables significantly correlated with the dependent variables were initially considered in the initial models. To avoid multicollinearity, explanatory variables with correlations greater than 0.7 were excluded. The significance level in all cases was set at *p* ≤ 0.05.

## 3 Results

Table 1 shows the overall sperm characteristics. Data for β‐galactosidase, GPX5, cathepsin B and ALP were only available from a small portion of the ejaculates. Several variables (15) were not normally distributed (Shapiro–Wilks test, *p* < 0.05). Therefore, a log_10_ transformation was applied to fructose, SOD, β‐galactosidase and GPX5 concentrations, as well as damaged acrosomes (%), early apoptosis (%), %DFI (%) and %HDS (%), the square root to protein and citrate concentrations and the arcsine function to Acro (%), sHOST (%), sORT (%), viability (%) and mitochondrial activity (%). Table 2 shows results on reproductive success after PCAI.

**TABLE 1:** Characteristics of ejaculates.

Variable	*n*	Mean ± SD
Protein (mg/mL)	104	41.92 ± 20.341
Citrate (mg/dL)	105	167.8 ± 89.930
Fructose (mg/dL)	99	4.3 ± 1.850
Zinc (μg/dL)	102	4298.31 ± 1575.856
SOD (U/mL)	98	1.93 ± 2.925
Beta‐Galactosidase (nmol/min/mg)	75	15.89 ± 27.453
GPX5 (log10 ng/mL)	50	166.06 ± 88.768
Cathepsin B (RFU)	69	21244.0 ± 3315.76
ALP (U/mL)	18	5.33 ± 2.910
pH	94	7.43 ± 0.082
AF	103	16.6 ± 5.49
Acro (%)	104	91.2 ± 8.88
TMot (%)	104	91.08 ± 5.241
PMot (%)	104	64.41 ± 8.592
Fast spermatozoa (%)	104	65.20 ± 14.878
Intermediate spermatozoa (%)	104	22.12 ± 8.974
Slow spermatozoa (%)	104	3.77 ± 3.145
Static spematozoa (%)	104	8.91 ± 5.241
VCL (μm/s)	104	68.25 ± 10.951
VSL (μm/s)	104	30.80 ± 5.274
VAP (μm/s)	104	47.89 ± 8.321
LIN (%)	104	45.47 ± 6.483
STR (%)	104	64.85 ± 8.198
WOB (%)	104	70.13 ± 4.209
ALH (μm)	104	2.50 ± 0.295
BCF (Hz)	103	6.58 ± 0.381
sHOST (%)	101	73.7 ± 6.27
sORT (%)	96	80.2 ± 7.61
Viability (%)	104	90.81 ± 6.167
Damaged acrosomes (%)	104	12.23 ± 7.739
Mitochondrial activity (%)	104	90.59 ± 4.693
Early apoptosis (%)	102	9.90 ± 4.443
cPARP_MFI	102	11.53 ± 0.330
PARP1_MFI	102	12.22 ± 0.338
PAR_MFI	102	12.65 ± 0.439
cPAR/PARP	102	0.94 ± 0.027
PAR/PARP1	102	1.04 ± 0.032
DFI (%)	102	4.54 ± 6.383
HDS (%)	102	7.65 ± 1.680

*Note:* Values are mean ± SD (standard deviation). *n*: Sample size.

**TABLE 2:** Reproduction success characteristics.

Variable	*n*	Mean ± SD
AUF (%)	92	95.4 ± 10.31
AFR (%)	92	94.3 ± 11.10
ATB	92	20.18 ± 2.029
ABA (%)	92	87.75 ± 5.607

*Note:* Values are mean ± SD (standard deviation). *n*: Sample size.

Abbreviations: ABA, average born alive (%); AFR, average farrowing rate (%); ATB, average total born; AUF, average ultrasound fertility (%).

The association between variables (Table 3) was assessed by Pearson correlation (*r*) or Spearman (ρ; non‐normally distributed variables: AUF, AFR and cathepsin B). Only significant *r* or ρ values are shown in Table 3 (*p* < 0.05).

**TABLE 3:** Significant correlation coefficients for reproductive success characteristics (*p* < 0.05).

Fertility outcomes	Semen variables; fertility outcomes	*n*	Pearson *r*; Spearman *ρ*	*p*‐value (bilateral)
AUF (%)	Damaged acrosomes (log_10_%)	92	−0.240	0.021
	Average farrowing rate (%)	92	0.855	< 0.001
AFR (%)	Damaged acrosomes (log_10_%)	92	−0.244	0.019
	Average ultrasound fertility (%)	92	0.855	< 0.001
ATB	Damaged acrosomes (log_10_%)	92	−0.304	0.003
ABA (%)	Protein (sqrt mg/mL)	91	0.273	0.009
	Fructose (log_10_ mg/mL)	86	0.243	0.024
	Cathepsin B (RFU)	58	0.257	0.029
	cPARP (MFI)	89	−0.295	0.005
	PAR (MFI)	89	−0.209	0.049

*Note:* Spearman ρ was estimated for AUF, AFR and cathepsin B; Pearson *r* was calculated in the rest of the cases. *n*: Sample size.

Abbreviations: ABA, average born alive (%); AFR, average farrowing rate (%); ATB, average total born; AUF, average ultrasound fertility (%).

Table 4 presents the significant regression models obtained for predicting ATB and ABA (%) from models that include several explanatory variables that have previously demonstrated a significant correlation with these reproductive success variables and are not strongly correlated among themselves.

**TABLE 4:** Significant regression models (*p* < 0.05).

Response variable	*n*	Explanatory variables	B	SE	*p*	95% CI for B		Beta	*R* ^2^	Δ*R* ^2^	Δ*R* ^2^ *p*‐value
						LL	UL				
ATB	92	Intercept	22.280	0.720	< 0.001	20.849	23.712		0.093	0.093	0.003
		Damaged acrosomes (log_10_%)	−2.111	0.696	0.003	−3.495	−0.728	−0.304			
ABA (%)	82	Intercept	114.092	20.173	< 0.001	74.552	122.618		0.089	0.089	0.006
		cPARP (MFI)	−4.894	1.749	0.006	−8.322	−1.466	−0.299			

*Note:* *n*: sample size.

Abbreviations: 95% CI for B, 95% confidence interval for the estimate; ABA, average born alive (%); ATB, average total born; B, regression coefficient estimate for the explanatory parameters; LL and UL, lower and upper limits for CI; *R* ^2^, coefficient of determination; SE, standard error for the estimate; Δ*R* ^2^ *p*–value, degree of significance of such change; Δ*R* ^2^, change in *R* ^2^ when the last explanatory variable was included in the model.

No significant model was found for AUF and AFR.

The model for ATB was significant (F_1,90_ = 9.191; *p* = 0.003) when damaged acrosomes (log_10_%) were included as the only explanatory variable. However, the coefficient of determination was low (*R* ^2^ = 0.093); only 9.3% of the variation in ATB was explained by this model.

For the ABA, cathepsin B (69 observations) was not considered as independent variable because only a small number of data are available. However, a high *r* value was obtained for PAR and cPARP (*r* = 0.708; *p* < 0.001), therefore only cPARP, with more intense correlation with ABA (Table 3), was considered as explanatory variable in the initial model, together with protein and fructose concentration. The model was significant (*F*_1,80_ = 7.830; *p* < 0.001) but with a low coefficient of determination (*R* ^2^ = 0.089); this model explained only 8.9% of the variation in ABA.

In both models, the negative estimators B (−2.111 ± 0.696 and −4.894 ± 1.749, respectively) indicated that an increase in acrosomal damage or cPARP was associated with a decrease in litter size or ABA respectively.

## 4 Discussion

This study shows that the evaluation panel in stud centers could be expanded to predict reproductive outcomes; however, given the current strict quality controls for sperm quality, any increases are small. In our case, fertility parameters were like those reported and considered satisfactory in modern sow farms (Freyer 2018). It is possible that the evaluated variables would have a higher predictive power in cases of lower fertility (other sow lines or during summer stress).

This study revealed significant association of sperm acrosomal damage (a “classic” parameter for sperm evaluation) with the fertility variables and the litter size. Previous studies have linked the acrosomal reaction to fertility in different species (Bernecic et al. 2021; Pampiglione et al. 1993); in pigs, a weak negative association has been found between acrosomal damage, assessed by microscopy, and the farrowing rate (Gadea et al. 2004; Galli and Bosisio 1988). It is not surprising that the association found in our study was similarly weak, since only ejaculates with less than 20% acrosomal damage were used as part of the selection criteria (Ausejo‐Marcos et al. 2024). It is possible that using flow cytometry, with higher precision than typical evaluations by microscopy, enabled the discovery of this relationship, even with good‐quality semen doses.

Another important finding was the association between SP parameters, including protein, fructose and cathepsin B levels and the number of piglets born alive. Although only part of the samples could be assessed for SP, this finding is still relevant. Seminal plasma components interact with spermatozoa during ejaculation, influencing motility, capacitation, transport, survival, and longevity, as well as through modulation of the female genital tract (González‐Cadavid et al. 2014; Mogielnicka‐Brzozowska and Kordan 2011; van den Berg et al. 2024). Considering SP proteins, a more detailed analysis could help refine this relationship, given their diverse roles. Indeed, no significant correlation was found between the enzymes SOD, β‐galactosidase and GPX5 with reproductive outcomes. The results for cathepsin B deserve more attention, since higher levels have been previously associated with low‐quality ejaculates (De Lazari et al. 2019); in contrast, our results show a positive association with ABA that could be related to the use of only good quality ejaculates which may mask the negative association. Furthermore, only a small amount of data was available for this variable which could explain a result that may not be real. In the case of fructose, this metabolite supports sperm motility and fertilisation in boars (Jones and Connor 2000; Tsujii et al. 2006) and it could be a biomarker of the good performance of the male reproductive tract (specifically, the seminal vesicles).

Whereas many studies have identified DNA fragmentation as a critical factor decreasing reproductive success, from mouse (Fernández‐Gonzalez et al. 2008) to human (Schulte et al. 2010), and even in rainbow trout (Pérez‐Cerezales et al. 2010), our study did not find significant correlations for SCSA variables. Interestingly, PAR and cPARP parameters related to the activation of PARP‐1 and to the activation of apoptotic pathways (Pascal 2018) respectively, were negatively correlated with the number of piglets born alive. Sperm chromatin is a relevant factor in sperm fertility and offspring viability, but its structure complicates its analysis in pigs (Gosálvez et al. 2011). Therefore, there is an interest in new biomarkers in this species and the PARP‐1 system could be a promising one (Lacalle et al. 2024).

Following the correlation analysis, the multivariate analysis delivered only two models, with limited predictive power. Fertilisation is a complex process, and sperm quality is just one of the many factors involved (Gadea 2005). Indeed, identifying the association and potential predictive value of new biomarkers was the primary objective of this study. For example, membrane biochemical activity, mitochondrial membrane potential, osteopontin and GPX5 (Michos et al. 2021), bacterial diversity and profile (Zhang et al. 2020) and epigenetic modifications such as differentially methylated regions (DMRs) have been proposed for this purpose (Pértille et al. 2021). Acrosomal damage evaluated by flow cytometry and cPARP levels could be added to this growing list and its relationship with fertility tested in a fully controlled experiment. Indeed, fertility and prolificacy are strongly influenced by sow‐related factors: housing (Verdon et al. 2015), weaning–oestrus interval (Bortolozzo et al. 2023), hormonal activity (Geisert et al. 2020) and lifetime performance (Koketsu et al. 2020).

In conclusion, fertility and prolificacy showed significant associations with several boar sperm parameters although the predictive value of these associations was low. These results are not unexpected when minimum sperm quality standards are met. Nevertheless, our study, starting from an ample set of variables, proposes potential new biomarkers for discriminating among good‐quality semen samples.

## Author Contributions

M.V.F., B.M., R.A.‐M., F.M.‐P., N.M.: conceptualisation. R.A.‐M., B.G.‐G., C.S.‐Ú., F.M.‐P., S.M.‐J., N.M., A.V.‐C., W.F.H., C.Á.H.: methodology. R.A.‐M., S.M.‐J., F.M.‐P., N.M., A.V.‐C.: investigation. M.T.T., S.M.‐J., A.V.‐C.: data curation. M.T.T., M.V.F.: writing – original draft preparation. R.A.‐M., M.T.T., M.V.F., S.M.‐J., C.S.‐Ú., B.G.‐G., F.M.‐P., A.V.‐C., N.M.: writing – review and editing. M.V.F., B.M.: supervision. M.V.F.: project administration. M.V.F., B.M., R.A.‐M, C.S.‐Ú: funding acquisition. All authors have read and agreed to the published version of the manuscript.

## Conflicts of Interest

The authors declare no conflicts of interest.

## Data Availability

The data that support the findings of this study are available from the corresponding author upon reasonable request.
